# Alterations of gut microbiota in gestational diabetes patients during the second trimester of pregnancy in the Shanghai Han population

**DOI:** 10.1186/s12967-021-03040-9

**Published:** 2021-08-26

**Authors:** Yao Su, Hong-Kun Wang, Xu-Pei Gan, Li Chen, Yan-Nan Cao, De-Cui Cheng, Dong-Yao Zhang, Wen-Yu Liu, Fei-Fei Li, Xian-Ming Xu

**Affiliations:** Department of Obstetrics and Gynecology, Shanghai General Hospital, Shanghai Jiao Tong University School of Medicine, No. 100 of Haining Road, Hongkou District, Shanghai, 201600 China

**Keywords:** Gut microbiome, Gestational diabetes mellitus, Glucose, Metabolism, Pregnancy

## Abstract

**Background:**

The causes of gestational diabetes mellitus (GDM) are still unclear. Recent studies have found that the imbalance of the gut microbiome could lead to disorders of human metabolism and immune system, resulting in GDM. This study aims to reveal the different gut compositions between GDM and normoglycemic pregnant women and find the relationship between gut microbiota and GDM.

**Methods:**

Fecal microbiota profiles from women with GDM (n = 21) and normoglycemic women (n = 32) were assessed by 16S rRNA gene sequencing. Fasting metabolic hormone concentrations were measured using multiplex ELISA.

**Results:**

Metabolic hormone levels, microbiome profiles, and inferred functional characteristics differed between women with GDM and healthy women. Additionally, four phyla and seven genera levels have different correlations with plasma glucose and insulin levels. Corynebacteriales (order), *Nocardiaceae* (family), *Desulfovibrionaceae* (family), *Rhodococcus* (genus), and Bacteroidetes (phylum) may be the taxonomic biomarkers of GDM. Microbial gene functions related to amino sugar and nucleotide sugar metabolism were found to be enriched in patients with GDM.

**Conclusion:**

Our study indicated that dysbiosis of the gut microbiome exists in patients with GDM in the second trimester of pregnancy, and gut microbiota might be a potential diagnostic biomarker for the diagnosis, prevention, and treatment of GDM.

**Supplementary Information:**

The online version contains supplementary material available at 10.1186/s12967-021-03040-9.

## Introduction

Pregnancy is characterized by increased insulin resistance and immune tolerance of the fetus and placenta. These induce metabolic and immunological changes throughout the pregnancy [1–3]. These physiological alterations may result in the development of gestational diabetes mellitus (GDM) [3]. GDM is defined as any abnormal glucose regulation onset or first recognized during pregnancy [4]. GDM is one of the most common complications during pregnancy. The incidence rate is about 17.5% in China and shows a gradually increasing trend [5]. GDM is becoming a significant threat to maternal and neonatal health, including cardiovascular disease, obesity, and pre-eclampsia in the mother [6], and fetal macrosomia, premature birth, shoulder dystocia, congenital malformations, and other issues in the baby [7]. Even though the glucose regulation of GDM often normalizes shortly after delivery, the increased risk of type 2 diabetes is 40% in the 10–15 years following pregnancy [8, 9]. The pathogenic factors are still not apparent. These may be caused by insulin resistance and pancreatic B cell secretion defects resulting in genetic or environmental effects. Recently, more studies have found that both patients with type 2 diabetes and patients with GDM have an imbalance of gut microbiota [10].

Microbes that reside in the human gut are recognized as one of the most important contributors to the host metabolism and immune system health [11]. Koren et al. found that gut microbiota changes heavily from the first to the third trimester of pregnancy, causing an increase of genus diversity and a decrease of richness, with the increased abundance of Proteobacteria and Actinobacteria and a decreased quantity of *Faecalibacterium* in the third trimester [12]. These changes may lead to metabolic dysfunctions, for example, GDM during pregnancy [13]. Other studies have found that in the third trimester, women with GDM showed a higher abundance of Actinobacteria, *Collinsella*, *Rothia*, and *Desulfovibrio* than the healthy group [3]. However, studies on the gut microbiota of GDM have shown opposing conclusions; either no differences among different groups or with an increased quantity of Firmicutes and reduced Bacteroidetes and Actinobacteria [14] or a decreased quantity of the *Faecalibacteria* compared with women who were normoglycemic [15]. Other articles showed the opposite results: a decline in the *Faecalibacteria* in GDM [3]. Some examinations indicated the relationship between the gut microbiome and the development of GDM [16, 17].

However, due to the limitation of the cross-sectional study design and the small sample size, the exact mechanisms leading to these significant changes in dominant bacteria are still unclear. Many factors affect the composition of gut microbiota. Studies have shown that regional differences, dietary habits, and varying gestational weeks can affect the microbiome. To better acknowledge the composition of gut microbiota and the potential influence on the etiology of GDM in the Asian population, it is vital to know the composition of the gut microbiome when giving a GDM diagnosis to women. This study aims to find the different compositions of gut microbiota between patients with GDM and healthy individuals in China when giving a GDM diagnosis and indicate the relationship between biomarkers and bioinformatics of pregnant women. The functions between the gut microbiome and molecular substance metabolism were also inferred, which would be beneficial when conducting deeper research on the mechanisms of GDM.

## Methods

### Study population and sample collection

From September 2019 to June 2020, pregnant women with a 75 g oral glucose tolerance (OGTT) in their second trimester (24–28 weeks) referred to Shanghai General Hospital, Shanghai Jiao Tong University School of Medicine, were selected. The women collected their feces at home and brought it to the hospital on the morning of blood collection. If there were no feces on that day, the women were allowed to delay the collection for one day at most after blood collection. A total of 53 cases were divided into two cohorts: 21 individuals with GDM (according to the OGTT diagnosis standard: FPG ≥ 5.1 mmol/L, 1hPG ≥ 10.0 mmol/L, or 2hPG ≥ 8.5 mmol/L) [18], and 32 were allocated to the control group. Inclusion criteria: (1) patients were Shanghai residents and had a typical diet for the Songjiang District; (2) patients did not have diabetes or impaired glucose tolerance before pregnancy. Exclusion criteria: (1) multiple births; (2) diabetes, hypertension, thyroid disease, gastrointestinal or cardiovascular disease before pregnancy; (3) use of assisted reproductive technology; (4) antibiotic use in the previous 2 months; (5) active smokers. All participants provided written informed consent before enrollment, and the Ethics Committee of the Shanghai General Hospital approved the research. The most common maternal characteristics were fasting plasma glucose (FPG) levels, one-hour plasma glucose (1hPG), two-hour plasma glucose (2hPG), fasting insulin levels (FINS), one-hour plasma insulin (1hPIN), two-hour plasma insulin (2hPIN), homeostatic model assessment for insulin resistance (HOMA-IR), triglyceride (TG), total cholesterol (TC), high-density lipoprotein (HDL), and low-density lipoprotein (LDL). These values were obtained from their medical records. On the day the blood was drawn, the serum aliquots were collected and stored at − 80 °C. Fresh feces were also collected and stored in the freezer at − 80 °C until DNA extraction.

### DNA extraction

A frozen aliquot (200 mg) from each fecal sample was suspended in 250 µL of guanidinium thiocyanate, 0.1 M Tris (pH 7.5), and 40 µL of 10% *N*-lauroyl sarcosinate. Total bacterial genomic DNA samples were extracted from 53 specimens using Qiagen QIAamp DNA Stool Mini Kits (Qiagen, California, USA). NanoDrop 2000 (Thermo Scientific, USA) was used to detect the concentration of the extracted DNA. The samples that did not meet the detection standards were removed.

### 16S rRNA amplicon pyrosequencing

The v3–v4 variable regions of 16S rRNA were specifically amplified by a polymerase chain reaction (PCR) with the forward primer 338F (5ʹ-ACTCCTACGGGAGGCAGCAG-3ʹ) and the reverse primer 806R (5ʹ-GGACTACHVGGGTWTCTAAT-3ʹ), where the barcode is an eight-base sequence unique to each sample. PCR reactions were performed in triplicate with a 20 μL mixture containing 4 μL of 5 * FastPfu Buffer, 2 μL of 2.5 mM dNTPs, 0.8 μL of each primer (5 μM), 0.4 μL of FastPfu Polymerase, and 10 ng of template DNA. Reactions were conducted under the following conditions: initial denaturation (95 °C, 2 min), 25 cycles at 95 °C (30 s), annealing at 55 °C (30 s), extension at 72 °C (30 s), and a final extension at 72 °C (5 min), and 10 °C until halted by the user. PCR products were extracted from 2% agarose gels and purified using the AxyPrep DNA Gel Extraction Kit (Axygen Biosciences, Union City, CA, USA) according to the manufacturer’s instructions and quantified using QuantiFluor™-ST (Promega, USA). The PCR products were sequenced, and a database was established by an Illumina MiSeq instrument (Illumina, San Diego, California, USA) at SHBIO Corporation (Shanghai, China).

### Sequence analysis

Raw FASTQ files were demultiplexed and quality filtered using QIIME (version 1.9.1) with the following criteria. Default parameters: operational taxonomic units (OTUs) were clustered with a 97% similarity cutoff using UPARSE (version 7.1 http://drive5.com/uparse/), and chimeric sequences were identified and removed using UCHIME. The taxonomy of each 16S rRNA gene sequence was analyzed by RDP Classifier (http://rdp.cme.msu.edu/) against the SILVA (SSU123) 16S rRNA database using a confidence threshold of 70%.

### Bioinformatics and statistical analysis

The QIIME (version 1.9.1) and R packages were mainly used for gut microbiota sequence analyses. Alpha diversity indices, such as the Chao richness estimator, Shannon Diversity index, observed species, and coverages, were calculated using the OTUs table in QIIME to investigate gut microbiota community richness. Beta diversity was measured by Bray–Curtis and unweighted and weighted UniFrac Distance [19]. Beta diversity analysis was applied to evaluate the structural variation of microbial communities, including principal coordinates analysis (PCoA) based on UniFrac Distance matrix analysis and visualized via non-metric multidimensional scaling. The total microbial composition difference of the two groups was indicated by PERMANOVA (permutated analysis of variance) [20]. The Tax4Fun [21], an available source R package obtained from the SILVA dataset (version 132), was used for functional profiles. Pattern recognition analysis based on a forward feature selection combined with linear discriminant analysis (LDA) was performed using the R version 3.5.1 [21]. The unique and shared OTUs among samples were illustrated by Venn diagrams using the R package “Venn Diagram.” Taxa relative abundance at all levels was statistically compared between the two groups by Kruskal–Wallis tests from the R statistics package. The microbiota–microbiota correlation network was constructed using the SparCC algorithm [22] and visualized with Cytoscape version 3.4.0 [23].

Normal distributed continuous variables were illustrated by mean ± standard deviation and analyzed by *t*-tests, while non-normal distributed continuous variables were reported as median with interquartile ranges (Q1–Q3) and analyzed using the Wilcoxon signed-rank test or the Mann–Whitney U test conducted with SPSS version 23.0 (SPSS Inc., Chicago, IL, USA). *P* < 0.05 was considered significant. The McNemar Chi-square test, Pearson’s Chi-square test, or Fisher’s exact test were applied for dichotomous variables. LEfSe (LDA effect size) [24] with a P-value cutoff of 0.05 and LDA score cutoff of 2 was utilized to obtain the differential taxa and functions between the two groups. The different taxa were analyzed using LEfse to identify discriminative microbial markers between the GDM and control groups. Then, lists of different taxa ranked by random forests in order of feature importance were determined over 100 iterations. The discriminative taxas were input for the random forest classifier to predict the discrimination between the GDM and control. The receiver operating characteristic (ROC) curve was obtained (SPSS v.19.0) to display the constructed models. The area under the ROC curve (AUC) was used to designate the ROC effect. Spearman’s rank correlation was used for correlation analysis between different groups.

## Results

### Characteristics of pregnant women

The characteristics of the participants are presented in Table 1. The pre-pregnant body mass index (BMI) markers and gestational ages showed no difference between the two groups. While the pregnant women diagnosed with GDM were younger, the age and pre-BMI did not affect gut microbiota composition. Only the GDM disease status had a significant influence on microbiota by PERMANOVA analysis (Additional file 1: Table S1). The women diagnosed with GDM had higher FPG (*P* = 0), 1hPG (*P* = 0), 2hPG (*P* = 0.024), FINS (*P* = 0.016), 2hPIN (*P* = 0.016), and HOMA-IR (*P* = 0.008) than women who were normoglycemic. The two groups had similar Hb1ACs, 2hPINs, TGs, TC, HDL, and LDL.

**Table 1:** Maternal characteristics and biochemical data

Maternal characteristics and biochemical variables	GDM (n = 21)	Control (n = 32)	*P* value
Age (years)	28.7 ± 3.42	31.5 ± 4.56	0.019*
Pre-BMI (kg/m^2^)	21.7 (19.96–23.47)	22.6 (18.95–25.25)	0.94
Gestational age (weeks)	25.3 (25–26)	25.6 (25–26)	0.32
Fasting glucose (mmol/L)	5.0 ± 0.43	4.7 ± 0.23	< 0.01**
1 h glucose (mmol/L)	9.15 (8.18–10.25)	7.35 (6.13–8.77)	< 0.01**
2 h glucose (mmol/L)	7.27 (6.51–8.08)	6.33 (5.77–7.29)	0.024*
Hb1AC (%)	5.01 (4.85–5.1)	4.84 (4.5–5.1)	0.091*
Fasting insulin (μU/L)	58.07 (40.1–66.4)	46.94 (32.45–47.58)	0.016*
1 h insulin (μU/L)	573.3 (301.6–728.4)	359.27 (198.1–480.2)	0.016*
2 h insulin (μU/L)	383.31 (183.1–574.8)	301.55 (199.2–368.0)	0.363
HOMA-IR	1.88 (1.25–2.25)	1.41 (0.91–1.43)	0.008**
Triglyceride (mmol/L)	2.31 ± 0.68	2.62 ± 0.82	0.171
Total cholesterol (mmol/L)	6.02 ± 1.14	6.11 ± 0.82	0.741
HDL (mmol/L)	1.86 ± 0.38	2.0 ± 0.39	0.199
LDL (mmol/L)	2.99 ± 0.79	2.97 ± 0.73	0.926

Clinical characteristics, biochemical and hormonal variables of GDM and normoglycemic pregnant women at 24–28 weeks gestation are presented as mean ± SEM when normally distributed or median with 25–75th interquartile range when non-normal distributed. Statistically significant difference between the GDM and normoglycemic women group are highlighted (* P < 0.05, ** P < 0.01). HOMA-IR, HOMA-IR = FPG (mmol/L), FINS(μU/mL)/22.5

### A survey of the average daily diet of the pregnant women

In Table 2, the average daily diet is classified according to cereals, meat and poultry, Seafood, Diaries intake and vegetable and fruit. We also studied the daily total energy intake of pregnant women. There was no statistical difference in diet between the two groups.

**Table 2:** A survey of the average daily dietary intake of pregnant women

	GDM (n = 21)	Control (n = 32)	P value
Total energy intake, kcal/day	2018 (1466–2260)	2037 (1646–2447)	0.482
Cereals intake, g/day	321 (350–500)	350 (350–500)	0.284
Meat and poultry intake, g/day	170 (25–238)	160 (125–125)	0.544
Seafood intake, g/day	108 (25–125)	103 (25–125)	0.614
Dairies intake, g/day	256 (125–350)	267 (125–350)	0.816
Vegetable and fruit intake, g/day	313 (125–500)	397 (350–500)	0.125

Data are expressed as mean(Q1–Q3) by the Mann–Whitney U Test. Dietary composition mainly includes five types: cereals, meat and poultry, Seafood, Dairies and vegetable and fruit

### Altered gut microbiota in women with GDM

**Fig. 1:**
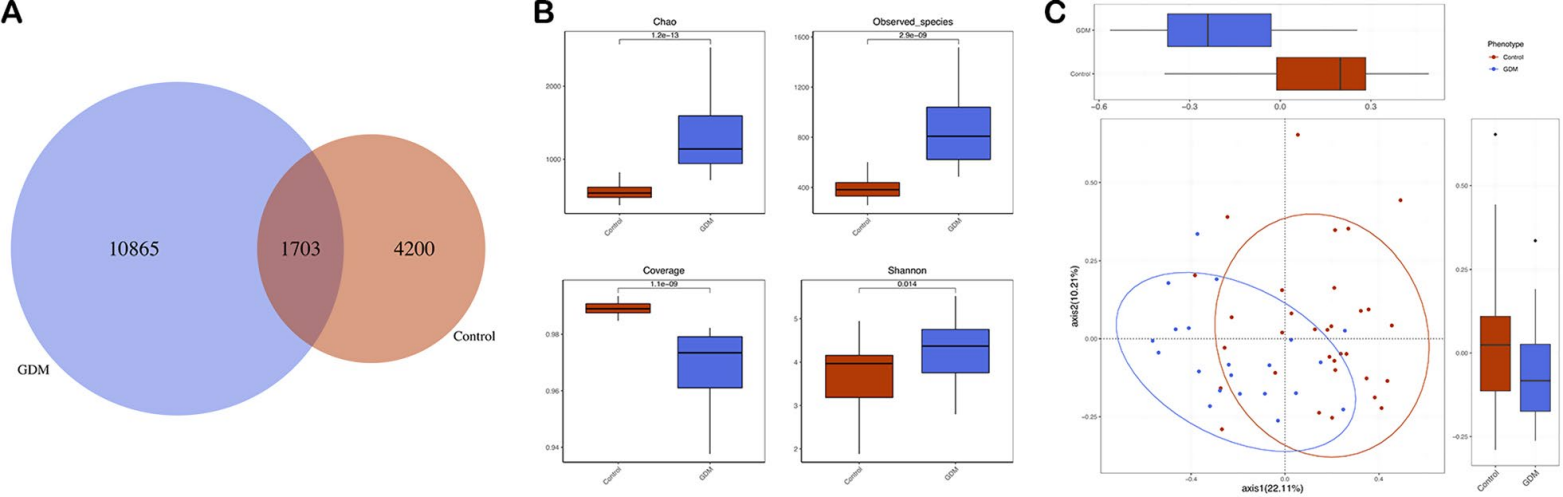
The richness and diversity of the gut microbiota in gestational diabetes mellitus and healthy control groups. **A** Venn diagram of gestational diabetes mellitus and control groups. The gestational diabetes mellitus group had more operational taxonomic units than the healthy control group. Alpha diversity **B** was calculated with QIIME2 v. 2018.2 based on the sequence similarity at the 100% level, including Chao estimator observed species coverage and Shannon index. The gestational diabetes mellitus group showed higher alpha diversity than the control. Principal coordinates analysis based on the weighted UniFrac matric **C** showed that the overall fecal microbiota composition differed between gestational diabetes mellitus and the control group. Each point represents one sample of gestational diabetes mellitus (red, n = 21), control (blue, n = 32) pregnant women. The distance among different samples reflects the comparability of the two cohorts

In the GDM group, the total number of OTUs was 12,568, with 10,865 unique OTUs, while the control cohort had 5903 OTUs, with 1,703 OTUs shared between the two groups (Fig. 1A). The analysis of alpha diversity (Fig. 1B) indicated women with GDM presented with a higher richness (Chao index, *P* = 1.2e−13) and higher diversity (Shannon index, *P* = 0.014) than detected in the control group. The microbial community also differed significantly for weighted UniFrac Distance between the two groups (Fig. 1C).

The top ten phyla in the two groups were shown (Additional file 2: Figure S1A) and the predominant genera in both the GDM and the control cohorts were Firmicutes (GDM: 52%, Control: 59%). There was no difference in Firmicutes between the two groups. Bacteroidetes (GDM: 41%, Control: 22%, *P* = 0.001) increased in the GDM group, while the other four (Proteobacteria, GDM: 5%, Control: 11%, *P* = 0.013; Actinobacteria, GDM: 0.8%, Control: 4.5%, *P* = 0; Verrucomicrobia, GDM: 0.2%, Control: 1.5%,* P* = 0.002; and Tenericutes, GDM: 0.1%, Control: 0.6%, *P* = 0.009) decreased and had significant differences compared with the control group. To further explore the altered gut microbiota in pregnant women with GDM, the sixteen genera were also shown (Additional file 2: Figure S1B). The predominant genus found in both groups was *Bacteroides* (GDM: 36%, Control: 11%). This increased in the GDM group and was statistically significant (*P* = 0.001). At the genus level, GDM showed a significantly higher abundance of *Incertae sedis* (*P* = 0.037), *Citrobacte*r (*P* = 0.02), *Parabacteroides* (*P* = 0.006), and *Fusicatenibacter* (*P* = 0.022). The healthy controls had significantly high levels of *Escherichia shigella* (*P* = 0.019), *Ruminococcaceae UCG014* (*P* = 0.003), *Eubacterium coprostanoligenes group* (*P* = 0.008), *Christensenellaceae R7 group* (*P* = 0.001), *Subdoligranulum* (*P* = 0.006), *Akkermansia* (*P* = 0.001), *Collinsella* (*P* = 0.005), *Lachnospiraceae UCG004* (*P* = 0.021), *Rhodococcus* (*P* = 0), and *Desulfovibrio* (*P* = 0.008). These findings revealed dysbiosis of the gut microbiota among women with GDM.

**Fig. 2:**
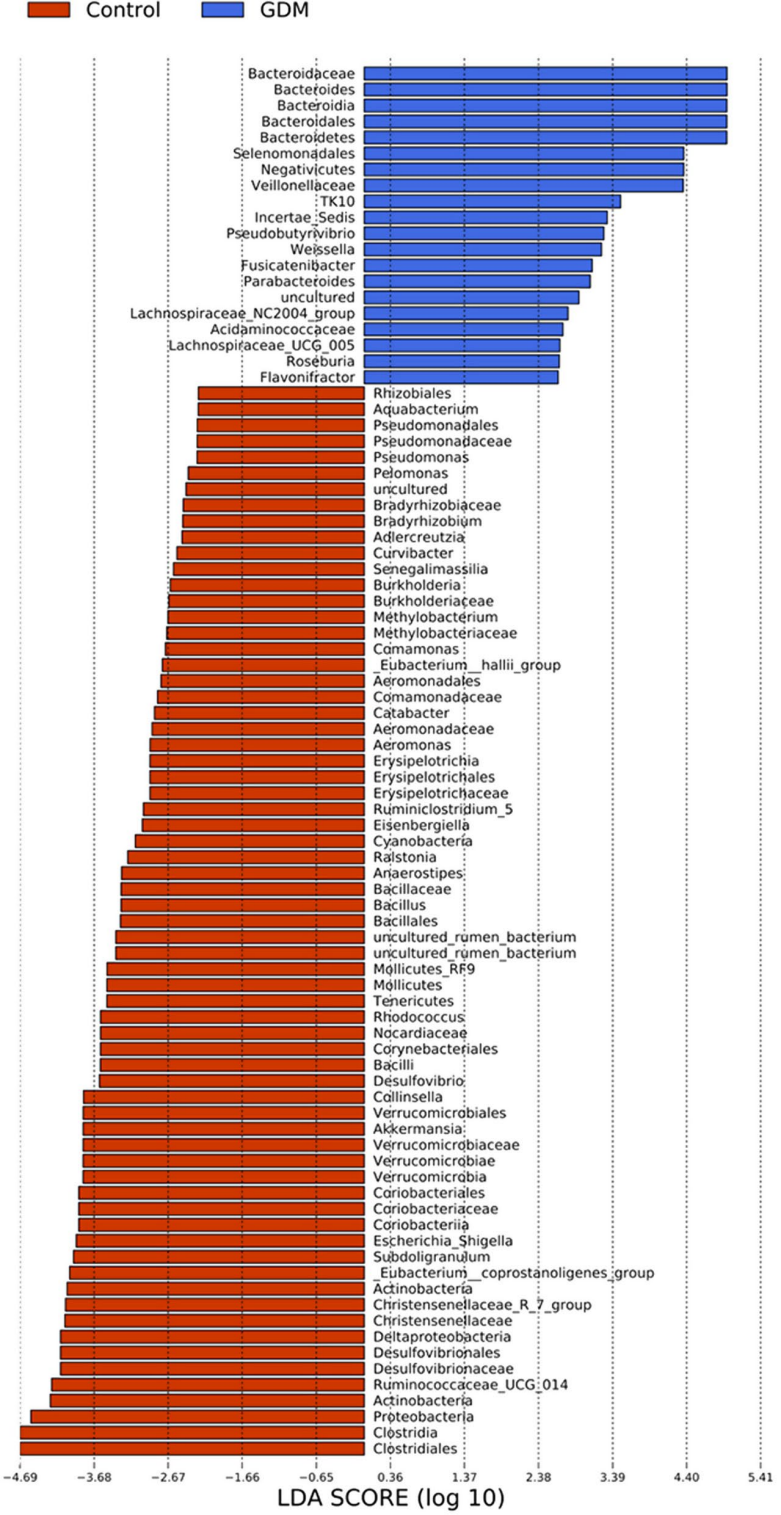
87 Differentially abundant taxa between the two groups. These different genera from phylum to genus were identified by linear discriminant analysis (LDA) using LEfSe. Linear discriminant analysis bar D: blue: gestational diabetes mellitus; red: healthy control

Eighty-seven differentially abundant taxa were found between the two groups using linear discriminant analyses, all of which had a log_10_ LDA score > 2 (Fig. 2). At the phylum level, the relative abundance of Bacteroidetes was higher in the GDM group while the Proteobacteria, Actinobacteria, Verrucomicrobia, Tenericutes*,* and Cyanobacteria were higher in the control group. At the order level, the relative abundance of *Corynebacteriales* was higher in the control group. At the family level, *Nocardiaceae* and *Desulfovibrionaceae* were higher in the control group. At the genus level, the relative abundance of *Bacteroides*, *Weissella*, *Fusicatenibacter*, *Parabacteroides*, *Roseburia*, *Flavonifractor,* etc., were higher in the GDM group, while the relative abundance of *Akkermansia*, *uncultured rumen bacterium*, *Ruminococcaceae UGG 014*, *Collinsella*, and *Escherichia shigella* was higher in the control group.

**Fig. 3:**
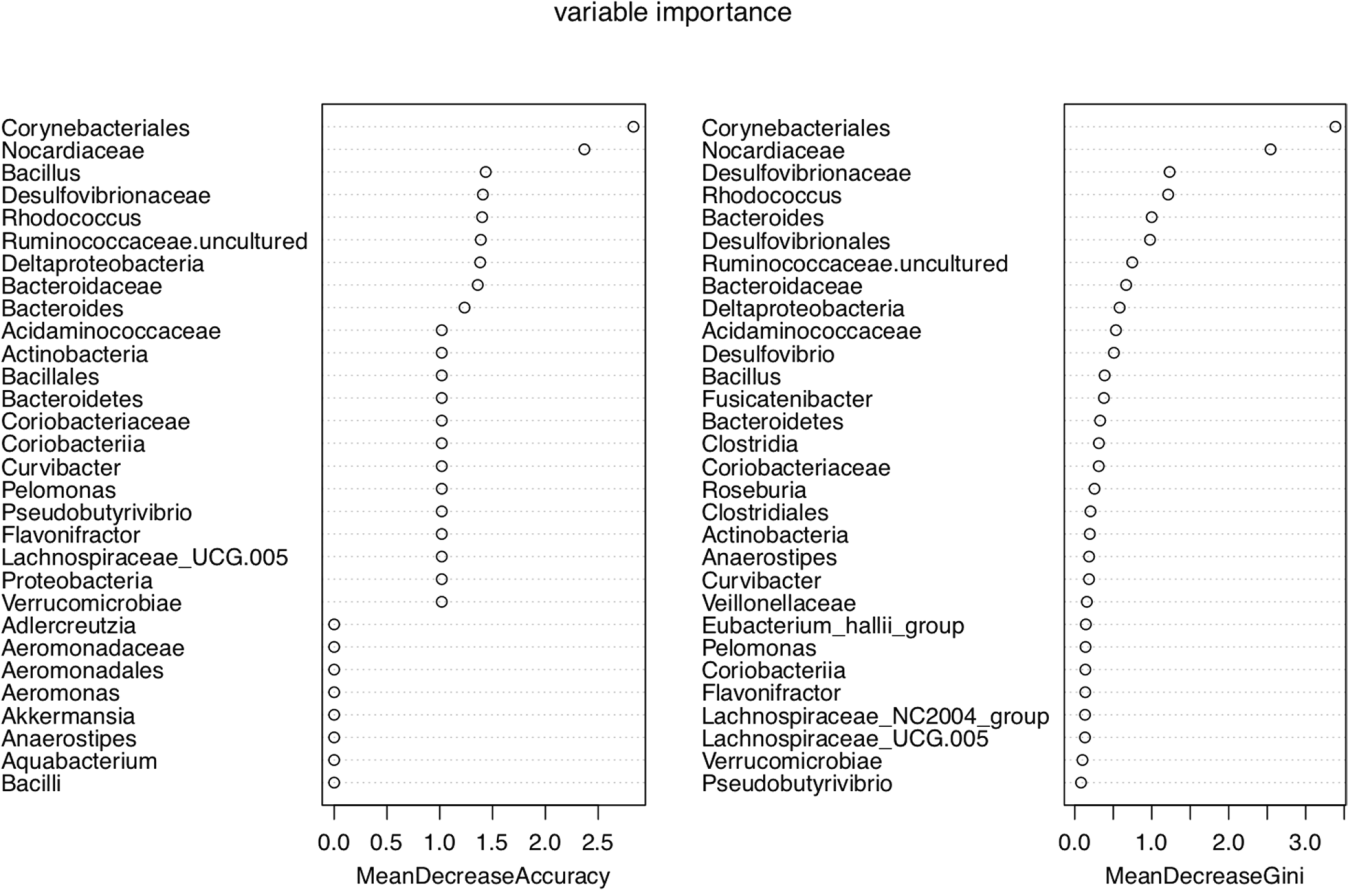
10-fold cross-validation with five repeats to evaluate the importance of taxa. Hollow points represent different genera. This figure revealed important bacterial classes as biomarker taxa to correlate with gestational diabetes mellitus

**Fig. 4:**
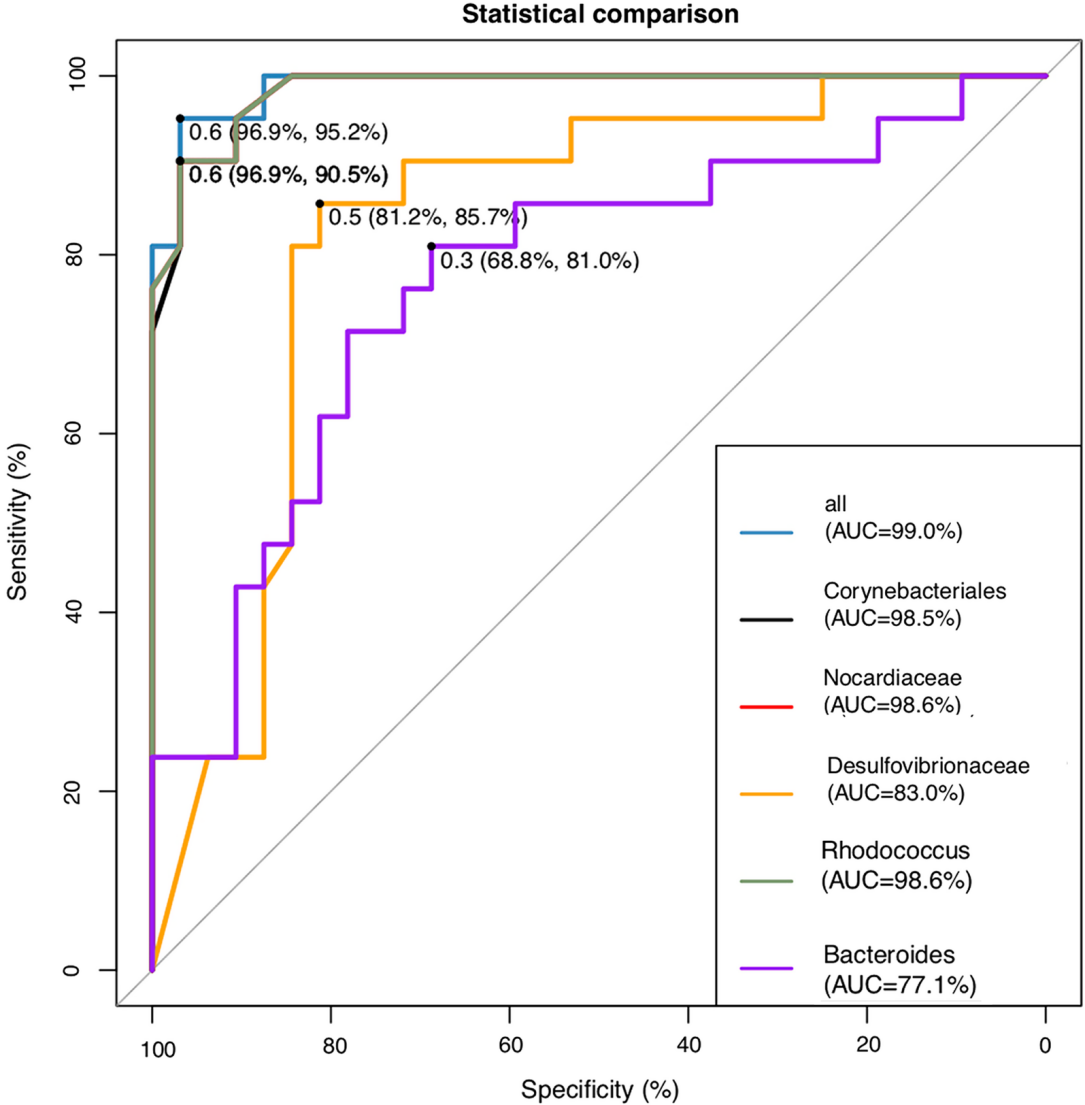
Random forest classifiers achieved an area under the curve analysis for a biomarker of gestational diabetes mellitus. Black line: *Corynebacteriales*, red line: *Nocardiaceae*, green line: *Rhodococcus,* orange line*: Desulfovibrionaceae,* purple line: *Bacteroides*, Blue line: the total five genera

A tenfold cross-validation was performed with five repeats to evaluate the importance of taxa to reveal important bacterial classes as biomarker taxa to correlate with GDM (Fig. 3). The minimum cross-validation error of 0.25 was obtained when using five important taxa, including Corynebacteriales (order), *Nocardiaceae* (family), *Desulfovibrionaceae* (family), *Rhodococcus* (genus), and *Bacteroides* (genus). Random forest classifiers achieved an AUC of 0.99 to detect patients with GDM (Fig. 4).

### Association of microbial composition with glycemic traits

**Fig. 5:**
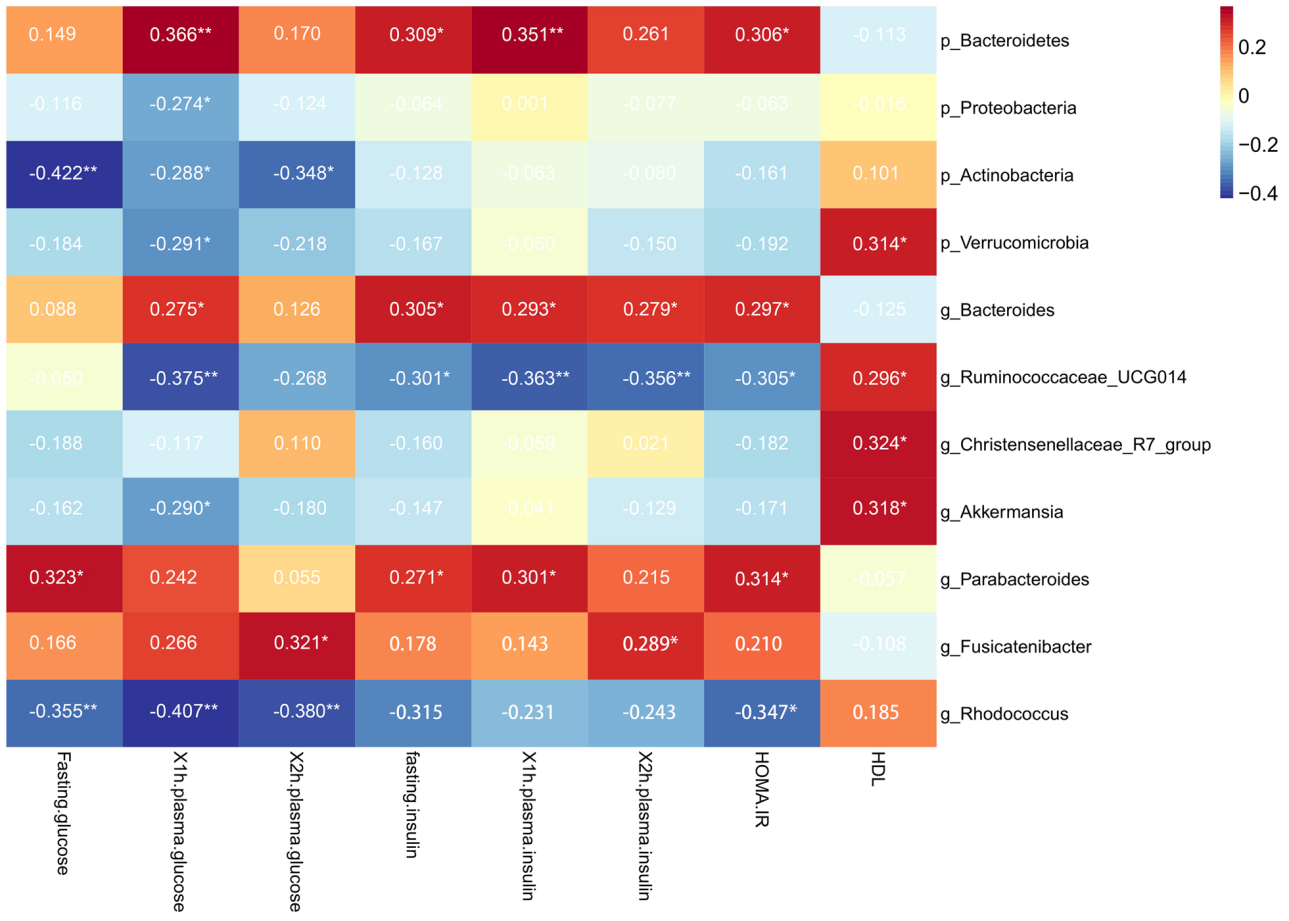
Gut microbiota abundance (phylum & genus) and their correlation with clinical characteristics and biochemical variables in gestational diabetes mellitus and the control. Heatmap of Spearman’s rank correlations between differential genera (LEfSe: *P* < 0.05 and linear discriminant analysis threshold value > 2) and clinical, biochemical variables. Bars that tend toward red and tend toward blue represent positive and negative correlations, respectively

Spearman’s correlation was used to identify deeper level of taxa associated with glycemic traits in pregnant women, regardless of their diabetic status, and found that the phylum Bacteroidetes was positively associated with 1hPG (*r* = 0.366, *P* = 0.007), FINS (*r* = 0.309, *P* = 0.024), 1hPIN (*r* = 0.351, *P* = 0.01), and HOMA-IR (*r* = 0.306, *P* = 0.026). *Proteobacteria*, Verrucomicrobia, and Actinobacteria were all negatively associated with 1hPG (*r* =  − 0.274, *P* = 0.047;* r* =  − 0.291, *P* = 0.034; *r* =  − 0.288, *P* = 0.036, respectively). Actinobacteria was also negative with FPG (*r* =  − 0.422, *P* = 0.002) and 2hPG (*r* =  − 0.348, *P* = 0.011). Verrucomicrobia was positive with HDL (*r* =  − 0.314, *P* = 0.024). Genus level revealed a negative association between *Ruminococcaceae UCG014* and 1hPG (*r* =  − 0.375, *P* = 0.006), FINS (*r* =  − 0.301, *P* = 0.028), 1hPIN (*r* =  − 0.363, *P* = 0.007), 2hPIN (*r* =  − 0.356, *P* = 0.007), and HOMA-IR (*r* = 0.305, *P* = 0.027), whereas it was positive with HDL (*r* =  − 0.296, *P* = 0.033). The increased relative abundance of *Incertae Sedis* genus was positively associated with higher FPG (*r* = 0.436, *P* = 0.001), 1hPG (*r* = 0.311, *P* = 0.023), and 1hPIN (*r* = 0.293, *P* = 0.033), while *Christensenellaceae R7 group* was only positive with HDL (*r* = 0.324, *P* = 0.019). The genus of *Akkermansia* was negative with 1hPG (*r* =  − 0.29, *P* = 0.035) and positive with HDL (*r* = 0.318, *P* = 0.022). HOMA-IR increased with the higher abundance genus of *Parabacteroides* (*r* =  − 0.314, *P* = 0.022) and decreased with higher *Rhodococcus* (*r* =  − 0.347, *P* = 0.011) (Fig. 5). Rhodococcus shows a strong negative correlation with 1hFG (R =  − 0.407).

### Inferred functional characters of the gut microbiota in GDM

**Fig. 6:**
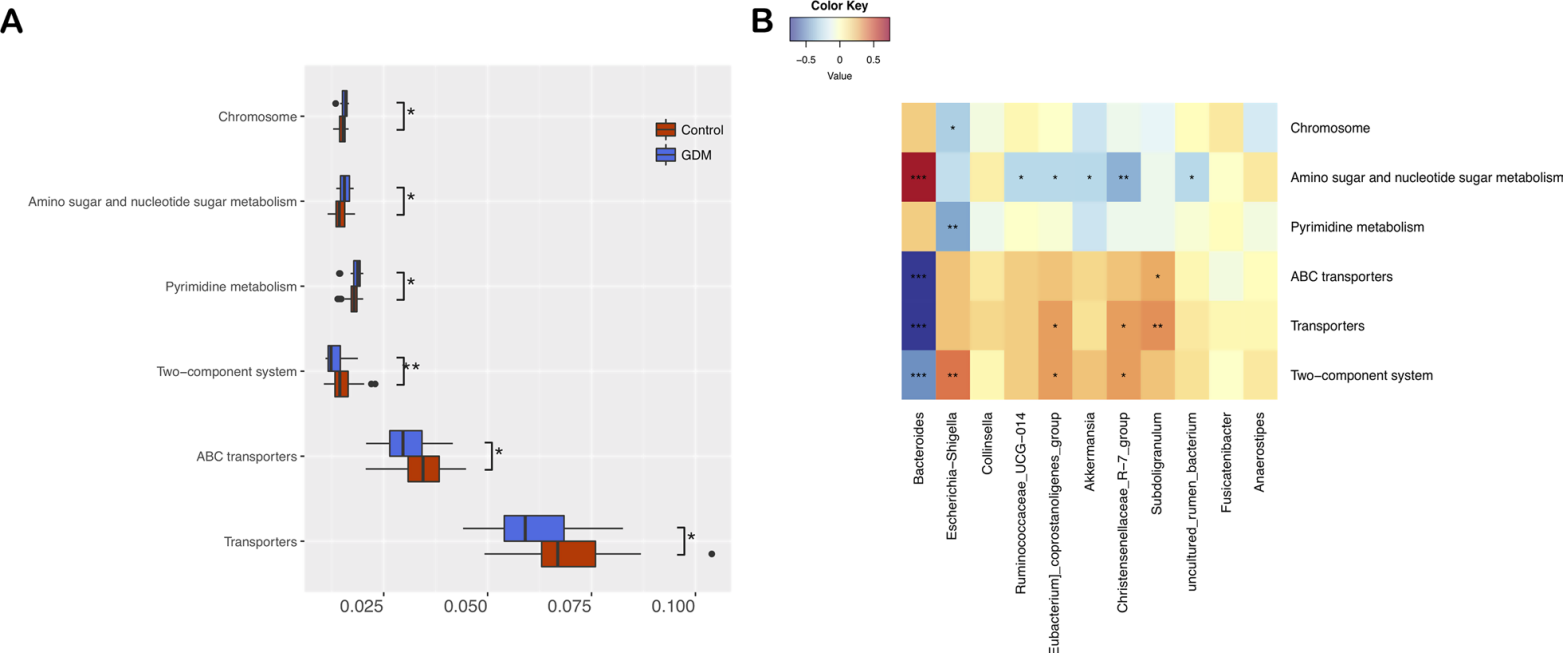
Functional analysis of the gut microbiota in healthy control and gestational diabetes mellitus groups. **A** Boxplot of differential functions between the control and gestational diabetes mellitus groups. The differential pathways were identified using Tax4Fun and LEfSe, *: *P* < 0.05, **: *P* < 0.01. LEfSe: linear discriminant analysis. **B** Heatmap of Spearman’s rank correlations between differential genera (LEfSe: *P* < 0.05 and linear discriminant analysis threshold value > 2) and differential pathways. Bars that tend toward red and tend toward blue represent positive and negative correlations, respectively

Six differential pathways were identified between the GDM and control groups by Tax4Fun and LEfSe (Fig. 6A). The predicted metagenomes for GDM depicted an enrichment of chromosomes, amino sugar and nucleotide sugar metabolism, pyrimidine metabolism, and a reduction of the two-component system, ABC transporters, and transporters. In the process of plotting correlations between differential genera and inferred metabolism pathways (Fig. 6B), significant positive connections were discovered between GDM-enriched *Bacteroides* genus and amino sugar and nucleotide sugar metabolism, as well as a negative association with the two-component system, ABC transporters, and transporter pathways. The other genus enriched in the control group (*Escherichia shigella*,* Eubacterium coprostanoligenes group*, *Christensenellaceae R7 group*, *Subdoligranulum*) were positively associated with the two-component system, ABC transporters, and transporter pathways. The genera of *Ruminococcaceae UCG014*, *Eubacterium coprostanoligenes group*, *Akkermansia*, and* Christensenellaceae R7 group* were all negatively associated with amino sugar and nucleotide sugar metabolism.

## Discussion

This study investigated the composition of the gut microbiome between women with GDM and women who were normoglycemic, comparing the different compositions of the two groups to find the connection between the microbiome and glucose metabolism during the second trimester of pregnancy. This study will provide a better understanding of the association between gut microbiota inferred functions and the metabolism of GDM. Ultimately, it will help in formulating scientific intervention measures from a comprehensive perspective.

The 16S rRNA gene was sequenced for the total bacterial DNA of stool samples from 21 women with GDM and 32 women who were healthy as the control group. These women, who were in the middle of their pregnancy (24–28 weeks), were randomly selected. Based on bioinformatic analyses of the GDM group, these women showed an increased richness and individual diversity (alpha diversity). Specifically, the phylum Bacteroidetes increased in GDM and increased *Bacteroides*, *Incertae Sedis*,* Citrobacter*,* Parabacteroides*, and *Fusicatenibacter* genus*.* These discoveries were similar to previous studies where *Parabacteroides* were significantly more enriched in women with GDM than in the healthy control group [18]. A few of the genus belonging to the Bacteroidetes phylum have been reported with dysbiosis among patients with GDM [18]. Patients who adhered to the dietary recommendations showed a better metabolic and inflammatory pattern and a significant decline in *Bacteroides* [16]. They also found that LPS inferred KEGG genes correlated with *Bacteroides*. LPS is reported to play an important pathogenic role in patients with diabetes. LPS originates from a species belonging to *Bacteroides* [25]. Additionally, *Bacteroides* is often associated with a high-fat, animal-based diet [26]. Our study found that the GDM group had more genus *Bacteroides,* which is negatively associated with HOMA-IR. *Bacteroides* and *Prevotella* have been recognized as contributors to insulin resistance and glucose intolerance [27].

The phylum Proteobacteria, Actinobacteria, and Verrucomicrobia were reduced in the GDM group, with *Escherichia shigella*,* Akkermansia*, *Ruminococcaceae UCG014*,* Christensenellaceae R7 group*, etc., decreasing. It has been reported that during normal pregnancies, gut microbiota maintained relative stability [15] or changed dramatically, such as the increased abundance of Proteobacteria and Actinobacteria, a decrease of butyrate-producing bacteria, a decline in bacterial richness, and with subject diversity (alpha diversity) [12].

When the relationships of metabolic traits and microbial taxa were investigated, it was found that *Akkermansia* is inversely correlated to 1 h plasma glucose and positively correlated with HDL, similar to a previous study. *Akkermansia* has previously been reported to be associated with improved metabolic health. It is also reported to be negatively associated with fasting plasma glucose and positively with insulin sensitivity. In a rodent study, adding *Akkermansia* probiotics improved glucose tolerance and insulin sensitivity [28–30]. However, *Akkermansia* has been connected to lower estimates of whole-body insulin sensitivity [3]. The genus *Christensenella* was recognized to be highly heritable and associated with low BMI [31]. An insufficient quantity of *Christensenella* has been linked to pre-diabetic health and is associated with increased acetate levels [32]. In a rodent model, germ-free transplantation of *Christensenella* showed a protective factor against weight gain [31]. In this study, though a significantly associated relationship with BMI was not found, the *Christensenellaceae R7 group* was positively associated with HDL.

*Corynebacterials* possess an atypical didermic cell envelope which belongs to Actinobacteria. In the study of gene expression of the bacteria found that more than half of the genes involved in the complex of Mycoloyl-arabinogalactan-peptidoglycan [33]. A study analyzed the gut microbime of 144 prediabetic subjects [34] and found that the relative abundance of *Nocardiaceae* and *Rhodococcus* was significantly increased. Studies have found that polycystic ovary syndrome (PCOS) is closely related to metabolic syndrome, especially insulin resistance [35]. In addition, Shermel B. Sherman et al. [36] found that the relative abundance of *Corynebacteriales*, *Nocardiaceae* and *Rhodococcus* in the intestine of PCOS pregnant sows increased significantly. *Desulfovibrionaceae* belongs to Gram-negative bacteria [37], which can induce low-grade persistent inflammation through lipopolysaccharide, leading to obesity and insulin resistance. Studies have found that mice with impaired glucose tolerance (IGT) increased body fat as well as the abundance of *Desulfovibrioceae.* In addition, *Desulfovibrioceae* is the key producer of bacterial endotoxins in obese animal models [38], or sulfate-reducing bacteria, by reducing sulfate to H2S, thereby destroying the intestinal barrier, leading to metabolic endotoxemia [39].

Microbial gene functions related to amino sugar and nucleotide sugar metabolism were higher in patients with GDM, especially the *Bacteroides* genus, which was positively associated with 1 h plasma glucose and HOMA-IR. Patients with GDM were characterized by enriched *Bacteroides* depleted *Ruminococcaceae UCG014, Akkermansia*, *Rhodococcus*, and *Lachnospiraceae UCG004*. *Bacteroides* have been studied would enhance host energy storage in di-associated mice [40]. *Bacteroides* secrete proteases on the brush surface of the absorbing cells. These proteases are similar to elastases and, when secreted in large quantities, degrade maltase and sucrase on the brush border without affecting alkaline phosphatase activity. They also concluded that brush border damage might occur from proteases secreted by *Bacteroides* in bacterial overgrowth syndromes [41]. Compared with non-GDM at their second and third trimester, there was a reduction in the relative abundance of SCFA-producing genus *Faecalibacterium, Ruminococcus, Roseburia, Coprococcus, Akkermansia, Phascolarctobacterium,* and *Eubacterium* in GDM women [42]. The SCFAs can combine with G protein-coupled receptors (GPCR) 41 and GPCR 43 to promote the secretion of peptide tyrosine tyrosine (PYY) and glucagon-like peptide (GLP)-1 from enteroendocrine cells [43]. This helps regulate insulin release and promote glucose metabolism. SCFAs also play a vital role in strengthening the intestinal barrier and decreasing inflammation and oxidative stress by activating the peroxisome proliferator-activated receptor (PPAR) pathway [44]. This study suggests that the disturbance of the intestinal microbiota may participate in the pathogenesis of GDM by regulating the host’s amino acid and carbohydrate metabolism, providing a new way to understand the basis of GDM.

In the αdiversity analysis of pregnant women's gut microbiome, pregnant women with GDM show higher Chao index, Observed-species, Shannon index and lower Coverage index, which is really different from previous studies. The reasons are as follows:

First, the gestation time of the study was different. We looked at the first to second trimester before OGTT testing.

Second, there are regional differences in the study population: the pregnant women included in our study were from China—Shanghai—Songjiang region, and the living environment and dietary habits of pregnant women in different regions may differ in the composition of microflora.

Third: the sample size of the study is different, and the detection results of different sample sizes will be different. Although we have tested more than 50 samples to reach the conclusion of this paper, we will expand the sample size and continue to study the bacterial composition of pregnant women and conduct dietary fiber intervention to study the changes of the bacterial community during the whole pregnancy.

A study by the Xiangya School of Public Health at Central South University in China found a similar finding: In early pregnancy (10 to 15 weeks of pregnancy), the intestinal microflora of pregnant women with GDM is mainly composed of Bacteroidetes, Firmicutes and Proteobacteria, Bacteroidetes is the dominant microflora of pregnant women with GDM and normal healthy pregnant women, and the abundance of Bacteroidetes is increased in pregnant women with GDM [45]. In addition, studies have also found that under the Western diet, Bacteroidetes are still the main dominant bacteria in GDM pregnant women and normal healthy pregnant women in the third trimester, and the abundance and alpha diversity of OTUs are also increased compared with postpartum [3].

Though this study found that these two patient groups had a similar dietary habit, many studies have shown that diet is one of the most important factors affecting gut microbiome composition. Recent studies have identified individual gut types that respond differently to particular diets [46]. According to the dominant bacteria, the population can be divided into three microbial enterotypes: *Bacteroides* (type B), *Prevotella* (type P), and *Ruminococcus* (type R) [47]. Most people belong to the first two intestinal types. These clusters seem to be independent of nationality, gender, age, and BMI but are determined by dietary habits [48]. The previous studies showed that high saturated fatty acid intake in the early postpartum stage decreased the abundance of Proteobacteria and Firmicutes. Dietary monounsaturated fatty acids helped increase the amounts of Firmicutes, Proteobacteria*,* and Bacteroidetes. The intake of vitamin A and vitamin D decreased the alpha diversity of the microbiome and increased the abundance of Proteobacteria [49]. It is necessary, given the strong association between gut microbiota and host location [50] as well as ethnicity [45], to carry out further verification on other pregnant women to evaluate whether the gut microbiota could be a predictor of GDM and guide pregnant women to accept reasonable and scientific intervention measures.

This prospective study is based on scientific principle, comparing the gut microbiome of pregnant women with GDM and healthy pregnant women during their OGTT period. It is pivotal to formulating the next intervention for women with hyperglycemia. However, several limitations of this study need to be addressed. First, the sample size was small, so it is not a good representation of pregnant women. Second, all the participants were from the same hospital. Different places need to be considered. Third, fecal samples were collected in the second trimester of pregnancy only. Thus, they would not convey all changes. In order to address these limitations and confirm the findings of this study, a multi-center, multi-point, vertical cohort research with gut microbiome analysis will be required.

## Conclusion

In conclusion, aberrant gut microbiome compositions in the second trimester of pregnant women, the markers of GDM, and the connection between the gut microbiome and plasma glucose were found. If confirmed by further large-sampled and well-designed research, these results of the gut microbiome dysbiosis might be involved in the pathogenesis of GDM. Additionally, these biomarkers might be potential predictors for GDM and would support individual prevention and intervention strategies for women with GDM. The inferred functions between the gut microbiome and molecular substances metabolism were useful in conducting more thorough research on the mechanisms of GDM.

## Supplementary Information

**Additional file 1: Table S1.** PERMANOVA results for the analysis of demographic characteristics associated with gut microbial community in 53 individuals based on BrayΓÇôCurtis distance.

**Additional file 2: Figure S1.** The composition of gut microbiome between the two groups in phylum and genus level. The top ten phyla **A** and sixteen genus **B** in the two groups are shown. There were significant differences in the composition of intestinal flora between the phylum and genus levels.

## Data Availability

We declared that materials described in the manuscript, including all relevant raw data, will be freely available to any scientist wishing to use them for non-commercial purposes, without breaching participant confidentiality.
