# Supplementary material for: Alterations of gut microbiota in gestational diabetes patients during the second trimester of pregnancy in the Shanghai Han population
Source: J Transl Med. 2021 Aug 26;19:366. doi: 10.1186/s12967-021-03040-9 (PMC8394568; doi:10.1186/s12967-021-03040-9)
Supplement: Supplementary file 1 — Additional file 1: Table S1. PERMANOVA results for the analysis of demographic characteristics associated with gut microbial community in 53 individuals based on BrayΓÇôCurtis distance. [file 12967_2021_3040_MOESM1_ESM.docx]

**Table S1 PERMANOVA results for the analysis of demographic characteristics associated with gut microbial community in 53 individuals based on Bray-Curtis distance**

| variable | Df | Sum of Sqs | F | P |
| --- | --- | --- | --- | --- |
| Group | 1 | 0.779 | 2.135 | 0.001 |
| Age | 1 | 0.381 | 1.045 | 0.355 |
| Pre-BMI | 13 | 4.830 | 1.018 | 0.329 |
