# Supplementary figures and images for: Alterations of gut microbiota in gestational diabetes patients during the second trimester of pregnancy in the Shanghai Han population
Source: J Transl Med. 2021 Aug 26;19:366. doi: 10.1186/s12967-021-03040-9 (PMC8394568; doi:10.1186/s12967-021-03040-9)

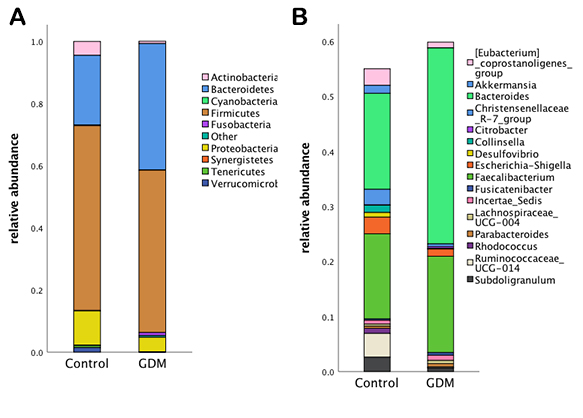

Supplement: Supplementary file 2 — Additional file 2: Figure S1. The composition of gut microbiome between the two groups in phylum and genus level. The top ten phyla A and sixteen genus B in the two groups are shown. There were significant differences in the composition of intestinal flora between the phylum and genus levels. [file 12967_2021_3040_MOESM2_ESM.jpg]
